# 

**Published:** 1852-12

**Authors:** 


[Several articles and bibliographical notices, intended for this number, have been crowded out by the Index, which occupies the last form. We ask the indulgence of those whose works are waiting notice.]

### NOTICE TO CORRESPONDENTS.

Communications and Books for notice should be addressed to the Editors, care of Messrs. Lindsay & Blakiston.

Letters, &c., connected with the *business affairs* of the Journal should be addressed to the Publishers.

Papers for publication must be received *before* the 20th of the month, or they cannot appear in the forthcoming number.

The following Journals have been received in exchange:

Medical News. Philadelphia.
Buffalo Journal.
L'Union Medicale.
New York Medical Gazette.
The Boston Medical and Surgical Journal. (Weekly.)
Stethoscope and Virginia Medical Gazette.
New Jersey Medical and Surgical Reporter.
Transylvania Medical Journal.
Nashville Journal of Medicine and Surgery.
Southern Medical and Surgical Journal.
New Hampshire Journal of Medicine.
British and Foreign Medico-Chirurgical Review.
The London Lancet. (Weekly, London.)
The Medical Times and Gazette. (Weekly, London.)
Dublin Medical Press.
London Journal of Medicine.
Edinburgh Medical and Surgical Journal.
Journal of Psychological Medicine.
London Pharmaceutical Journal.
Edinburgh Monthly Journal.
Journal of Pharmacy.
North Western Medical Intelligencer.
American Journal of Insanity.

---

### BOOKS RECEIVED.

Wilson on Syphilis. From Messrs. Blanchard & Lea.
Flint on Continued Fevers. From the Author.
Bond's Dental Medicine. From Messrs. Lindsay & Blakiston.
Green on Polypi of the Larynx, &c. From G. P. Putnam, N. Y.
Quekett's Lectures on Histology. From H. Bailliere, Esq.
Animal Chemistry, by Dr. Bence Jones. From the same.
Simon's Lectures on General Pathology.
Notes on Carpenter's Physiology, by Dr. Mackall.

---

The foreign correspondents of the Examiner will please direct their Exchanges and other communications to the care of Mr. Thos. Delf, No. 21 Paternoster Row, London, or Mr. H. Bossange, 21 Bis, Quai Voltaire, Paris.

